# The clinical applicability of a new automatic sugar analyser

**DOI:** 10.1155/S1463924681000072

**Published:** 1981

**Authors:** Klaus Dörner, Gisela Georgi, Gerd Liebezeit, Roger Dawson

**Affiliations:** 1 Universitäts-Kinderklinik Kiel Abtlg. Allgemeine Pädiatrie Kiel Germany; 2 Institut für Meereskunde Universität Kiel Kiel Germany


### The clinical applicability of a new automatic sugar analyser

**Klaus Dörner,* Gisela Georgi,**

*Universitäts-Kinderklinik, Kiel, Abtlg. Allgemeine Pädiatrie, Kiel, GFR.*

**and Gerd Liebezeit, Roger Dawson,**

*Institut für Meereskunde, Universität Kiel, Kiel, GFR.*

### Introduction

The clinical laboratory is regularly faced with the task of establishing the causes of non-diabetic melituria. These causes may be: physiological for neonates [1], caused by hereditary defects of carbohydrate metabolism [2], brought about by congenital or acquired deficiencies of disaccharidases [3], or an abnormal sugar excretion may be found with enteritis [4]. The necessary analysis is often approached qualitatively by using thinlayer chromatography [5, 6, 7]; this gives a quick result which is often sufficient. Ion exchange chromatography allows the quantitative determination of a range of carbohydrates in urine whereas enzymatic methods only measure single compounds.

Column chromatographic methods previously described for use on clinical materials [8, 9, 10] suffer from disadvantages; long analytical run-times are required and the corrosive orcinol-conc. sulphuric acid reagent is used to detect any non-reducing sugars. Recently a method [11] uses the non-corrosive Nanochrom II reagent, and is investigated here for its applicability to routine clinical analysis.

### Material and methods

### Instruments

The sugar analyser (Fluorimetric Sugar Module, Breda Scientific, Breda, The Netherlands) was used packed with DA X-4 20μ (Durrum Chemical Co., Palo Alto, USA) in the borate form treated according to Mopper [12]. Pressure measured at the pump head was below 30 bar (3MPa). A single channel flowthrough filter fluorimeter was used as the fluorimetric detector with a combined controller-integrator-recorder unit (Figure 1). The UV absorption was measured with a UV spectrophotometer UV-24 (Beckman, Munich, Germany). This company also supplied the glucose analyser for glucose and disaccharide determinations.

* *correspondence to this author*

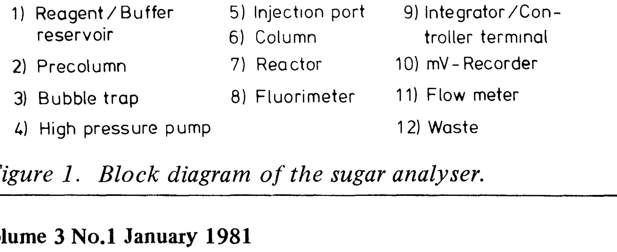

1) Reagent/Buffer reservoir

2) Precolumn

3) Bubble trap

4) High pressure pump

5) Injection port

6) Column

7) Reactor

8) Fluorimeter

9) Integrator/Controller terminal

10) mV-Recorder

11) Flow meter

12) Waste

*Figure 1. Block diagram of the sugar analyser.*

Ultrafiltration of samples was performed with a microfiltration apparatus 16538 (Sartorius, Göttingen, Germany) with a nitrogen overpressure of 10 bar (1MPa).

### Chemicals

All chemicals (Merck, Darmstadt, Germany) were of the highest purity grade. Enzymes and the test kit for galactose were from Boehringer, Mannheim, Germany (Catalogue No. 124273). Xylose was determined using the test kit "Determination of pentoses in urine" (Hoffmann-La-Roche, Grenzach, Germany Catalogue No. 0180). Nanochrom II (ethylenediamine, EDA) was obtained from Breda Scientific, Breda, The Netherlands. Standard solutions contained 0.7 mmoles/l glucose, xylose, galactose, fructose, mannose and sucrose respectively, and 0.18 mmoles/l of lactose.

Sugar solutions for addition experiments contained 50g sugar/l.

### Methods

The borate complexes of the carbohydrates were separated isocratically using a 0.7 M boric acid eluting buffer adjusted to pH 8.90 ± 0.01 with 8 N NaOH. The Column temperature was set to 80 - 81°C and a flow rate of 48 ml/h was used.

Carbohydrates are detected by the formation of a fluorophore in alkaline medium with EDA according to Honda et al [13]. As the reaction proceeds sufficiently rapidly only at elevated temperatures, it was possible to add the reagent (Nanochrom II) with a final concentration of 500 μl/l to the eluting buffer. After separation the sugars were reacted with EDA at 155° C for approximately 8 minutes. The absorbance of the resulting fluorophore was measured at two wavelengths, 345 and 450 nm. Details of the reaction conditions (effects of buffer molarity, pH, reaction temperature and time) are scheduled to be published elsewhere [11]. To eliminate possible effects of changes in the buffer/reagent composition, 50 μl of the standard solution was injected each day before analysis of samples. Quantification and peak identification was achieved by comparison of peaks and retention times with those of standard mixtures. The volume of the ultrafiltered samples (occasionally diluted 1:10 with doubly distilled water) was kept constant at 50 μl. For urine samples the running time was extended to 3 hours in order to elute uronic acids which, having longer retention times than glucose (90 min), would otherwise appear as a continuous base line increase during the next runs.

Quantitative evaluation of chromatograms was carried out by comparing peak heights multiplied by width at half peak height, because matrix effects present on the baseline posed difficulties for electronic integration (see later).

The enzymic determinations of glucose, xylose, and galactose were carried out according to the kit instructions, lactose and sucrose were determined according to Dörner [14].

Bacterial activity in urine was suppressed by the addition of 1% (v/v) thymol in isopropanol (10% w/v). For recovery experiments 9 ml of urine was made up to 10 ml with standard solution and doubly distilled water. Serum and spinal fluid were ultrafiltered and either analysed immediately or stored at -18° C.

### Results

### Choice of sugars investigated

Several urine samples from newly born infants and healthy adults were analysed to establish the "normal" pattern and to decide which sugars to investigate. A typical chromatogram with pure sugars is shown in Figure 2, and from neonatal urine in Figure 3. Lactose, xylose, galactose, and glucose were routinely found to be present with occassional occurence of mannose, fructose, and sucrose. With the exception of mannose and fructose chemical methods already existed in the authors' clinics and could be used for comparative purposes.

Samples of serum and spinal fluid from subjects without metabolic disorders contained glucose with only traces of other sugars as shown in Figure 4.

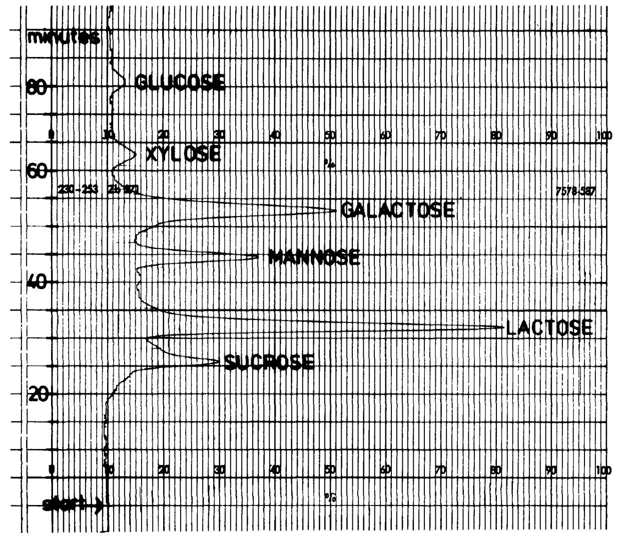

*Figure 2. Chromatogram of pure sugars; flow rate 52 ml $h^{-1}$ instead of 48 ml $h^{-1}$ as employed otherwise, thus resulting in shorter retention times.*

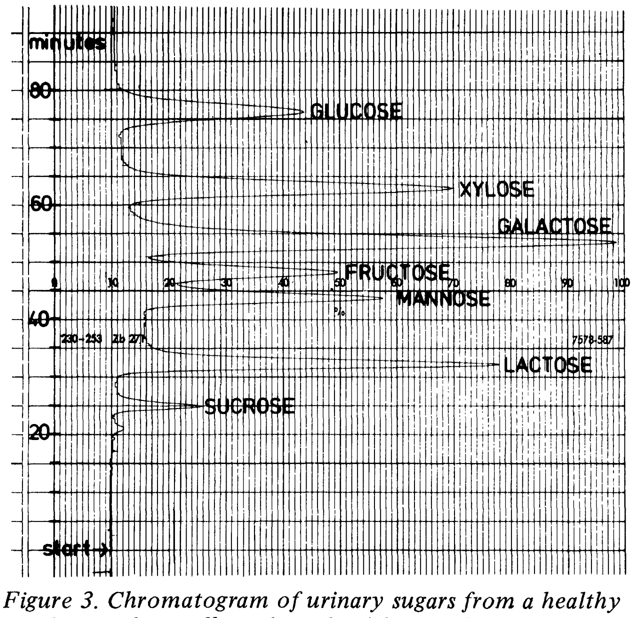

*Figure 3. Chromatogram of urinary sugars from a healthy newborn infant collected on the 4th day after birth; same running conditions as in Figure 2.*

### Linearity and sensitivity

The chromatographic method was tested with glucose in standard addition experiments as described above and found to be linear up to 2.5 mmoles/l (Figure 5). Higher concentrations were not tested. The reference methods were tested similarly and their linearity found to be satisfactory.

The detection limit is dependent on fluorimeter and recorder/integrator settings. For purposes of this study the smallest peak area that could be evaluated was considered to be 0.87 $cm^2$ corresponding to an integrator value of 100. For the reference methods, when an extinction difference of 0.005E was taken as being measurable, the detection limits were as shown in Table 1.

### Precision

Analytical precision was determined with 6 standard runs on two separate days. Results are given in Table 2. Coefficients of variation for sugars with long retention times are markedly smaller for the manual evaluation than for electronic integration. Repeated analysis (n = 6) of a newborn's urine gave coefficients of variation for lactose and galactose of 2.8% and 3.4% respectively. The retention times showed good reproducibility.

### Accuracy and recovery

An indication of accuracy was given on one sample by extrapolation of the recovery curve to y = 0 (Figure 5). The intercept concentration of 0.78 mmoles/l was in good agreement with the value of 0.68 mmoles/l determined on the initial sample without addition. Nine recovery experiments were carried out employing 4 different urines. Table 3 lists the concentration ranges together with recoveries found from chromatographic and conventional methods.

### Interference by preservatives and drugs, etc.

Addition of thymol in isopropanol was found to have no quenching effect on the fluorimetric signals.

Addition of sodium salicylate, sulfanilamide, ascorbic acid and Aldactone showed no quenching effect but rather a slight increase of the fluorimetric signal within the range of the standard deviation (cf. Table 3).

No bilirubin interference could be observed.

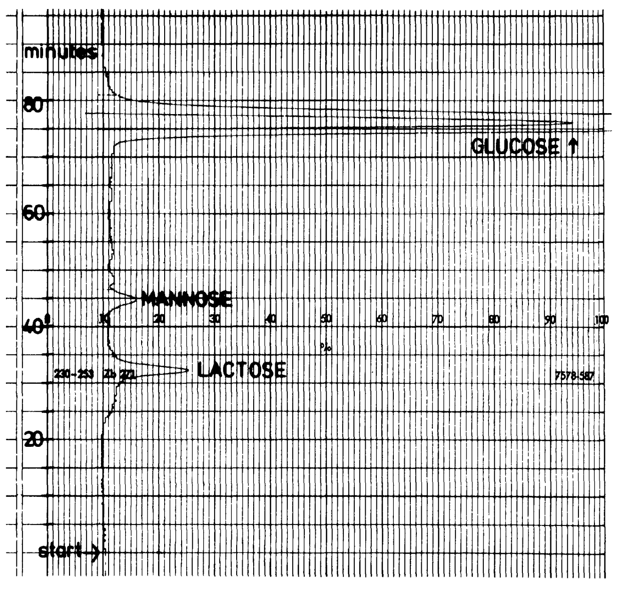

*Figure 4. Chromatogram from serum of an infant with biliary cirrhosis. The following concentrations were found: 3.84 mmoles/l glucose, 0.0618 mmoles/l mannose, and 0.0255 mmoles/l lactose. Sensitivity setting was higher than usual and the running conditions were as in Figure 2.*

Table 1. Detection limits of the methods used

| Sugar | Chromatography (mmoles/l) | Reference (mmoles/l) |
|---|---|---|
| Sucrose | 0.38 | 0.56 |
| Lactose | 0.02 | 0.56 |
| Galactose | 0.07 | 0.05 |
| Xylose | 0.17 | 0.43 |
| Glucose | 0.17 | 0.28 |

Table 2. Coefficients of variation of the peak areas (n = 6) and retention times

| Sugar | retention time [min] | | | coefficient of variation | |
|---|---|---|---|---|---|
| | mean | maximum | minimum | electronic | manual |
| Sucrose | 27.6 | 27.7 | 27.4 | 9.9 | 5.4 |
| Lactose | 36.2 | 36.3 | 36.0 | 4.9 | 4.4 |
| Galactose | 61.0 | 62.1 | 60.9 | 5.2 | 3.9 |
| Xylose | 72.1 | 72.9 | 71.8 | 10.7 | 3.1 |
| Glucose | 88.9 | 89.6 | 88.6 | 11.3 | 1.2 |

### Discussion

The analysis of complex mixtures of carbohydrates in physiological fluids has been carried out in the past mainly by paper [15] or thin layer chromatography [5, 6, 7]. For quantitative approaches ion exchange chromatography has certain advantages [8, 9, 10, 16, 17]. Long running times and the use of corrosive reagents have been largely overcome by recently developed techniques. Special resin treatment [12] and the use of the non-corrosive reagent Nanochrom II [11] have made it possible to carry out rapid determinations of carbohydrates. This reagent has the advantage over the non-corrosive Cu(II)-sodium bicinchionate-aspartic acid [18] in that non-reducing sugars are also detected. Since fluorimetric methods are sometimes sensitive to interferences from various external agents it is important to ascertain that the matrix (urine for example) does not influence the determination. No significant interference by sodium salicylate, ascorbic acid sulfanilamide, and aldactone could be established. As shown in Table 3 recoveries are generally good and superior to conventional methods. The ability of the built-in integrator to follow varying baselines and changing backgrounds encountered with urine samples was not entirely satisfactory with the basic integration program and a more complicated baseline tracking program is required. Since the peaks were symmetrical it was possible to use a manual method for peak area evaluation with good precision [Table 2]. The retention times showed little variance between runs. A slight shift towards shorter retention times was observed for urine samples due to their high salt content. This effect may be overcome by using a desalting step prior to analysis. This also serves to remove unwanted matrix substances.

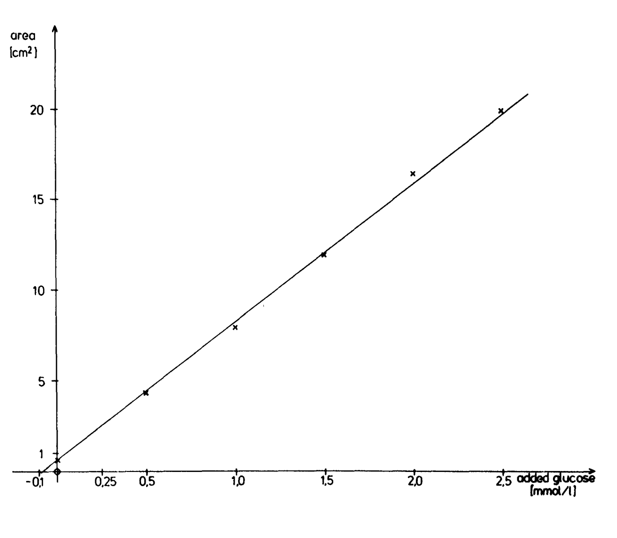

*Figure 5. Test of linearity with increasing addition of glucose standard to urine.*

Table 3. Results of recovery after the simultaneous addition of sucrose, lactose, galactose, xylose, and glucose

| Sugar | concentration range [mmoles/l] | mean recoveries [%] | |
|---|---|---|---|
| | | chromatography | conventional method |
| Sucrose | 3.65 - 5.84 | 96.2 | 81.2 |
| Lactose | 1.70 - 2.38 | 89.6 | 81.1 |
| Galactose | 4.16 - 9.71 | 83.4 | 85.4 |
| Xylose | 3.44 - 12.6 | 90.4 | 121.7 |
| Glucose | 5.73 - 12.49 | 99.2 | 79.3 |

Galactose/fructose and maltose/lactose were not separated with 0.7 molar buffer used. If fructosuria or maltosuria are suspected or if unsymmetrical peaks are detected, a second analysis should be carried out using a buffer of lower molarity. In the samples employed for this study these two compounds were not established to be present.

Uronic acids have retention times longer than glucose (90 min) and appear as extremely broad peaks. Their determination within two hours is possible by changing running parameters (higher buffer molarity, lower column temperature and addition of 0.03 to 0.06 mmoles/l sodium chloride).

Samples of urine, serum, and spinal fluid need only be ultrafiltered, no desalting or concentration was necessary. A decrease in separating ability with the analysis of physiological samples could not be detected. The observed yellow discolouration of the resin at the top of the column was probably due to protein and polysaccharide material of low molecular weight and had no marked effect on the separations.

The detection limits of the chromatographic system were good and can be decreased if necessary. Settings of the fluorimeter and recorder/integrator were adjusted to analyse normal urines without dilution.

REFERENCES

[1] Bickel, H., (1960) *Med. Probl. Paediat.* 6, 313
[2] Stanbury, J.B., Wyngaarden, J.B. and Frederickson, D.S. (1978), The metabolic basis of inherited disease, McGraw-Hill Inc. New York
[3] Erdin, H.P., Schweiz. (1978) *Rundschau Med.* (PRAXIS) 67, 334
[4] Lebenthal, E. (1975), *Ped. Clin. North America* 22, 757
[5] Young, D.S. and Jackson, A.J. (1970), *Clin. Chem.* 16, 954
[6] Kraffczyk, F., Helger, R. and Bremer, H.J. (1972), Clin. Chim. Acta 42, 303
[7] Menzies, I.S., Mount, J.N. and Sheeler, M.J. (1978), Ann. Clin. Biochem. 15, 65
[8] Jolley, R.L. and Freeman, M.L. (1968), Clin. Chem. 14, 538
[9] Seuffer, P., Voelter, W., Bauer, M. (1977), *J. Clin. Chem. Clin. Biochem.* 15, 663-668
[10] Jolley, R.L., Warren, K.S., Scott, Ch.D., Jainchill, J.L., Freeman, M.L. (1970), *Amer. J. Clin. Pathol.* 53, 793
[11] Mopper, K., Dawson, R., Liebezeit, G., Hansen, H.P. (1980). Anal.Chem.
[12] Mopper, K., (1978) *Anal. Biochem.* 87, 162
[13] Honda, S., Kakimoto, K., Sudo, K., Kakehi, K. and Takiura, K. (1974), *Anal. Chim. Acta* 70,133
[14] Dörner, K. (1977), *Europ. J. Ped.* 126, 45
[15] Menzies, I.S. (1973), J. Chromatogr. 81, 109, (1976) *Chromatographia* 9, 433
[16] Jandera, P., Churacek, J. (1974), J. Chrom. 98, 55
[17] Kennedy, J.F. (1974), *Biochem. Soc. Trans.* 2, 54
[18] Mopper, K., Gindler, E.M. (1973), Anal. Biochem. 56, 440

